# Neutrophil-to-lymphocyte ratio is a novel predictor of venous thrombosis in polycythemia vera

**DOI:** 10.1038/s41408-022-00625-5

**Published:** 2022-02-10

**Authors:** Alessandra Carobbio, Alessandro Maria Vannucchi, Valerio De Stefano, Arianna Masciulli, Paola Guglielmelli, Giuseppe Gaetano Loscocco, Francesco Ramundo, Elena Rossi, Yogendra Kanthi, Ayalew Tefferi, Tiziano Barbui

**Affiliations:** 1 FROM Research Foundation, Papa Giovanni XXIII Hospital, Bergamo, Italy; 2 Center Research and Innovation of Myeloproliferative Neoplasms (CRIMM), Department of Experimental and Clinical Medicine, Azienda Ospedaliera Universitaria Careggi, University of Florence, Florence, Italy; 3 Section of Hematology, Department of Radiological and Hematological Sciences, Catholic University, Fondazione Policlinico A. Gemelli IRCCS, Rome, Italy; 4 National Heart, Lung and Blood Institute, Bethesda, MD USA; 5 Division of Hematology, Department of Medicine, Mayo Clinic, Rochester, MN USA

**Keywords:** Myeloproliferative disease, Risk factors

## Abstract

We investigated the neutrophil-to-lymphocyte ratio (NLR) as a predictor of thrombosis in polycythemia vera (PV). After a median follow-up of 2.51 years, of 1508 PV patients enrolled in the ECLAP study, 82 and 84 developed arterial and venous thrombosis, respectively. Absolute counts of total leukocytes, neutrophils, lymphocytes, platelets, and the NLR were tested by generalized additive models (GAM) to evaluate their trend in continuous scale of thrombotic risk. Only for venous thrombosis, we showed that baseline absolute neutrophil and lymphocyte counts were on average respectively higher (median: 6.8 × 10^9^/L, *p* = 0.002) and lower (median: 1.4 × 10^9^/L, *p* = 0.001), leading to increased NLR values (median: 5.1, *p* = 0.002). In multivariate analysis, the risk of venous thrombosis was independently associated with previous venous events (HR = 5.48, *p* ≤ 0.001) and NLR values ≥5 (HR = 2.13, *p* = 0.001). Moreover, the relative risk in both low- and high-standard risk groups was almost doubled in the presence of NLR ≥ 5. These findings were validated in two Italian independent external cohorts (Florence, *n* = 282 and Rome, *n* = 175) of contemporary PV patients. Our data support recent experimental work that venous thrombosis is controlled by innate immune cells and highlight that NLR is an inexpensive and easily accessible prognostic biomarker of venous thrombosis.

## Introduction

Polycythemia vera (PV) is a myeloproliferative neoplasm (MPN) characterized by uncontrolled clonal proliferation of multipotent bone marrow progenitors, sustained by acquired genetic mutations in *JAK2* genes (*JAK2* V617F and exon-12 mutations). Typically, PV displays an elevated red-cell mass, usually along with leukocytosis and thrombocytosis [1]. Expansion of the mutated clone triggers an inflammatory response that influences the development of associated vascular complications and disease progression into myelofibrosis (MF) and acute leukemia (AL) [2, 3].

Thrombosis can be the first presenting sign preceding the diagnosis of MPN in 20% of cases, with a persistent risk during the follow-up, where the incidence is the highest in patients with PV and similar in essential thrombocythemia (ET) and primary myelofibrosis (PMF) [4, 5]. In a real-world experience of 1500 contemporary patients with PV [6], total major thrombosis rate was 2.62% patients per year, an estimate lower than that reported in the European Collaborative Low-dose Aspirin (ECLAP) trial [7] (4.4% patients per year) but comparable to the rate seen in the recent cytoreductive therapy in PV (CYTO-PV) study [8] (2.7% patients per year, venous and arterial thrombosis in 1.59% and 1.05% patients per year, respectively). Therefore, these estimates document that thrombosis remains a relevant unmet need in contemporary patients with PV both in conventionally defined low risk (age <60 and no prior vascular events) and high risk (age >60 years or prior thrombosis). The presence of cardiovascular (CV) risk factors may increase this risk, but given the inconsistency of the results [9, 10], when used on top of age and prior thrombosis, CV risk factors have not been included in a formal scoring system in PV, as they are in the International Prognostic Score for Thrombosis in Essential Thrombocythemia (IPSET thrombosis score) for ET [11].

The relationship between leukocytosis and thrombosis has been extensively investigated in several experimental studies [12, 13] based on the notion that in MPNs, chronic and subclinical systemic inflammation has a critical role in the pathogenesis of vascular events. However strong evidence in support of leukocytosis as an inflammatory biomarker potentially contributing to differentiate prognostic categories in PV is still missing. In a very recent meta-analysis [14], the association of leukocytosis with thrombosis was stronger in ET than in PV and exclusively related to arterial events, as shown in our previous analysis of ECLAP patients where time-dependent and not baseline leukocytosis was associated with myocardial infarction [15]. Other authors [16] found that the persistent leukocytosis in PV is linked to hematologic evolution rather than thrombosis.

Thus, no definitive prognostic role could be ascertained as regards leukocytosis, thus preventing it from being included in the thrombotic risk score of PV patients.

However, new data are emerging on the role of nonmyeloid inflammatory cells, such as T lymphocytes and monocytes in the process of immune thrombosis; in particular, experimental work has consistently showed that T-reg lymphocytes are involved in the regulation of the prothrombotic action of activated neutrophils in the process of fibrin formation and dissolution [17]. Based on this knowledge, the ratio between neutrophils to lymphocytes (NLR) might represent a synthesis of these two opposed actions in the thrombotic events and has a role as a prognostic marker of cardiovascular events in PV as it was shown in the general population and in a small series of patients with ET [18–20].

In the present work, we investigated whether NLR can identify PV patients at higher risk of both venous and arterial incident events by analyzing the ECLAP database that involved PV patients prospectively followed for a median of 3 years and includes a large number of formally validated arterial and venous thrombotic events.

## Materials and methods

### Patients

The present study included 1508 of the 1638 PV patients enrolled in the ECLAP study, selected on the basis of the availability of differential blood counts at baseline that allowed to calculate the NLR.

All patients with new and old PV diagnoses (according to Polycythemia Vera Study Group (PVSG) criteria) were included prospectively with no exclusion criteria with respect to age, therapy, or duration of disease. Treatment strategies had to comply with the recommendation of maintaining the hematocrit value at less than 0.45 and the platelet count at less than 400 × 10^9^/L. Data regarding clinical outcomes, treatments, and laboratory values during the prospective follow-up were recorded at follow-up visits at 12, 24, 36, 48, and 60 months.

The study protocol conformed to good clinical practices and to the Declaration of Helsinki on medical research in humans.

### Events

The primary endpoint was the incidence rate of arterial and venous thrombosis (myocardial infarction, nonfatal stroke, pulmonary embolism, major venous thrombosis, and minor thrombotic complications—including atypical cerebral or visual symptoms of ischemia, erythromelalgia, and thrombophlebitis), and major and minor thrombotic complications as defined above. These events were objectively diagnosed as previously reported [7].

### Statistical methods

Univariate analysis was performed to evaluate differences in proportions by the chi-square or Fisher exact tests where appropriate. Differences in continuous variables were tested using the nonparametric Wilcoxon rank-sum test.

In order to investigate the role of NLR in the time-to-thrombosis prediction, we overcame the traditional Cox proportional-hazard model, which restricts the log hazard ratio (HR) to be linear in the covariates, by applying the generalized additive models (GAM) [21]. GAM provide a flexible extension of the usual linear models and are capable of capturing nonlinear effects of predictors, therefore, they are useful in estimating trends of predictors as smooth functions of risk in continuous scale.

NLR values were dichotomized using the best threshold found according to Liu’s method. Thrombosis-free survival of the resulting two groups was compared using the Kaplan–Meier (KM) estimator and tested with the log-rank test.

Cox proportional-hazard models were applied for multivariable analyses. The Harrell’s C-statistic was calculated to measure the incremental discriminatory ability of a new predictor [22].

## Results

### Thrombotic events

During a median follow-up of 2.51 years, 166 thromboses (82 arterial and 84 venous) were recorded in 160 of 1508 patients (10.6%). Arterial thromboses were mostly cerebral, while venous thrombosis mostly affected lower extremities (DVT), with or without pulmonary embolism (PE) (Table 1).

**Table 1:** Types of 160 nonfatal thrombosis during follow-up (median follow-up time: 2.51 years).

	n/N = 1508 (%)
Nonfatal thrombosis	160 (10.6%)
*Arterial*	82 (5.4%)
Myocardial infarction	13 (0.9%)
Stroke	21 (1.4%)
Transient ischemic attack	32 (2.1%)
Peripheral arterial thrombosis	19 (1.3%)
*Venous*	84 (5.6%)
Deep vein thrombosis	35 (2.3%)
Pulmonary embolism	12 (0.8%)
Superficial thrombophlebitis	46 (3.1%)

### Patient characteristics stratified by thrombosis

Table 2 outlines patient characteristics at the time of study registration (baseline) with further stratification based on thrombosis events that occurred during the study follow-up. The analysis of risk factors assessed at the baseline, showed a marked difference between the groups. Arterial thromboses were associated with age (*p* = 0.001) and previous thrombosis (*p* = 0.003), especially if arterial (*p* < 0.001), and with the presence of at least one cardiovascular risk factor (*p* = 0.009). No association with baseline leukocytosis or the NLR was found. In patients with venous thrombosis, not age but history of thrombosis (*p* < 0.001) was also a risk factor for incident events, especially in cases with prior venous events (*p* < 0.001).

**Table 2:** Baseline characteristics according to occurrence and type of nonfatal thrombosis during follow-up.

	No thrombosis	Arterial thrombosis	Venous thrombosis
	*N* = 1,348	*N* = 82	*N* = 84
Sex, *n* (%)			
Female	558 (42.0%)	31 (37.8%)	40 (47.6%)
Male	770 (58.0%)	51 (62.2%)	44 (52.4%)
Age, years, median (IQR)	67.0 (57.6–74.3)	71.4 (63.2–76.3)^a^	67.6 (62.8–74.0)
≥65 years, *n* (%)	552 (40.9%)	46 (56.1%)^a^	33 (39.3%)
Previous thrombosis, *n* (%)	473 (35.1%)	42 (51.2%)^a^	51 (60.7%)^a^
Arterial	362 (26.9%)	37 (45.1%)^a^	22 (26.2%)
Venous	155 (11.5%)	11 (13.4%)	36 (42.9%)^a^
BMI, median (IQR)	24.9 (22.9–27.4)	25.3 (23.6–27.8)	25.6 (23.4–29.3)
Normal weight, *n* (%)	593 (51.1%)	33 (44.0%)	29 (43.3%)
Overweight, *n* (%)	443 (38.2%)	34 (45.3%)	26 (38.8%)
Obesity, *n* (%)	125 (10.8%)	8 (10.7%)	12 (17.9%)
At least one CV risk, *n* (%)	844 (62.6%)	63 (76.8%)^a^	52 (61.9%)
*Blood counts, median (IQR)*			
Hematocrit, %	0.5 (0.4–0.5)	0.5 (0.4–0.5)	0.5 (0.4–0.5)
Platelets, x10^9^/L	353.5 (247.5–500.0)	309.0 (229.0–482.0)	340.5 (239.5–486.5)
White blood cells, x10^9^/L	8.9 (6.7–12.4)	8.5 (6.2–12.0)	8.9 (7.0–11.9)
eutrophils, x10^9^/L	6.2 (4.3–9.3)	5.8 (3.8–9.1)	6.8 (4.6–9.3)^a^
Lymphocytes, x10^9^/L	1.7 (1.3–2.3)	1.7 (1.2–2.2)	1.4 (1.1–1.9)^a^
Neutrophils/Lymphocytes	3.5 (2.3–5.8)	3.1 (2.3–5.9)	5.1 (2.9–7.5)^a^
*Treatments, n (%)*			
Aspirin	456 (33.8%)	35 (42.7%)	29 (34.5%)
Phlebotomy	870 (64.5%)	51 (62.2%)	57 (67.9%)
Hydroxyurea	653 (48.4%)	38 (46.3%)	47 (56.0%)

*IQR* interquartile range, *BMI* body mass index, *CV* cardiovascular.

^a^Significant difference (i.e., *p*-value < 0.05) compared to “No thrombosis” group

Differently from arterial, venous complications significantly occurred for progressive increase of BMI (*p* = 0.041), and above all, it was greater in categories of patients with higher NLR values (*p* = 0.002), which resulted from a simultaneous increase of neutrophils (*p* = 0.002) and a decrease of lymphocytes (*p* = 0.001).

### Hazard ratio trends by absolute blood differential counts and NLR values

**Fig. 1:**
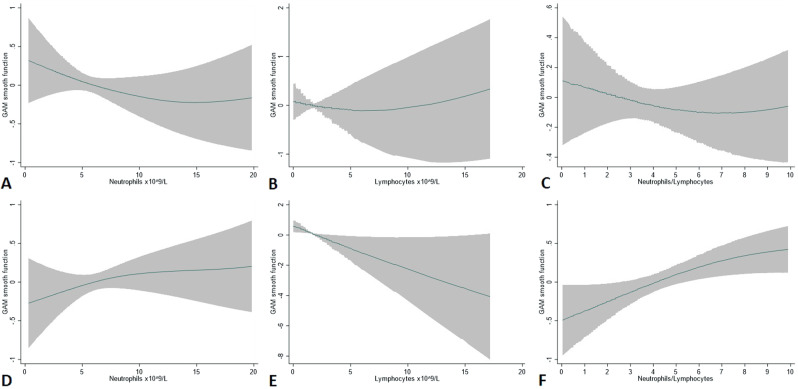
Generalized additive proportional-hazard models (GAM) for arterial and venous thrombosis. Generalized additive proportional-hazard models (GAM) for the prediction of arterial (**A**, **B**, **C**) and venous (**D**, **E**, **F**) thrombosis of absolute neutrophils, lymphocytes, and their ratio. The effect on the risk of arterial and venous thrombosis of neutrophils, lymphocytes, and their ratio is analyzed on a continuous scale by GAM smooth function with cubic splines. Hazard-ratio estimates (solid line) along with their 95% confidence intervals (gray area) are plotted in logarithmic scale.

Absolute values of total leukocytes, neutrophils, lymphocytes, platelets, and NLR were tested by GAM to capture in continuous-scale nonlinear relationships with thrombosis. A significant association between the risk of venous thrombosis and the groups of patients with progressive lower lymphocyte counts (*p* = 0.002) emerged, leading to increase the NLR values (*p* = 0.005) (Fig. 1D, E, F). The hazard ratio in logarithmic scale (log HR) of venous thrombosis was greater than 0 when absolute lymphocyte count was <2 × 10^9^/L, while it progressively decreased when the lymphocyte counts were higher, thereby supporting an opposite association with venous thrombosis. Of note, absolute neutrophil counts showed an opposite behavior.

Therefore, since the NLR integrates these two opposing trends, it was more representative of the association of lymphocyte and neutrophil counts with venous thrombosis than individual counts alone, where confidence intervals resulted wide at higher counts.

Conversely, neither absolute values of neutrophils and lymphocytes nor NLR were found associated with arterial events (Fig. 1A, B, C).

Total leukocyte and platelet counts were also tested, but they did not correlate with either venous or arterial thrombosis (data not shown).

### NLR as independent risk factor for thrombosis (Multivariable model)

In the multivariate model, adjusted for age, gender, previous thrombosis, and treatment at baseline (i.e., aspirin, phlebotomy and hydroxyurea (HU)), the risk of venous thrombosis was independently associated with previous venous events (HR = 5.43, *p* < 0.001) and NLR values ≥5, a figure representing the best cutoff resulted applying Liu’s method (HR = 2.14, *p* = 0.001, Table 3). In a sensitivity analysis, NLR effect for total venous thrombosis was not modified by excluding superficial venous thrombosis (HR = 2.87, *p* = 0.001, Table 3).

**Table 3:** Multivariable Cox proportional hazards models for the prediction of all venous thrombosis and deep vein thrombosis -with or without pulmonary embolism only.

Covariate	All venous thrombosis (*n* = 84)		DVT ± PE (*n* = 47)	
	HR (95% CI)	*P*-value	HR (95% CI)	*P*-value
Male sex	1.08 (0.69–1.67)	0.742	0.92 (0.50–1.68)	0.780
Age ≥65 years	0.96 (0.61–1.50)	0.847	1.44 (0.79–2.64)	0.234
Previous arterial event	0.91 (0.54–1.52)	0.714	1.12 (0.57–2.22)	0.737
Previous venous event	5.43 (3.48–8.46)	0.000	2.74 (1.41–5.29)	0.003
NLR ≥ 5	2.14 (1.38–3.30)	0.001	2.87 (1.56–5.27)	0.001
Aspirin	0.92 (0.56–1.49)	0.732	1.07 (0.56–2.06)	0.843
Phlebotomy	1.15 (0.72–1.84)	0.548	1.50 (0.76–2.93)	0.241
Hydroxyurea	1.28 (0.82–1.99)	0.273	1.43 (0.77–2.63)	0.255

*HR* hazard ratio, *CI* confidence interval, *NLR* neutrophil/lymphocyte ratio, *DVT* deep vein thrombosis, *PE* pulmonary embolism.

Older age (i.e., ≥65 years, HR = 1.82, *p* = 0.007) and previous arterial thrombosis (HR = 1.88, *p* = 0.007) retained the prognostic statistical significance for arterial thrombosis, together with an effect of CV risk factors of borderline significance (HR = 1.65, *p* = 0.052, Table 4).

**Table 4:** Multivariable Cox proportional hazards model for the prediction of arterial thrombosis.

Covariate	HR (95% CI)	*P*-value
Male sex	0.74 (0.48–1.15)	0.183
Age ≥65 years	1.82 (1.17–2.82)	0.007
Previous arterial event	1.88 (1.18–2.99)	0.007
Previous venous event	0.96 (0.51–1.83)	0.911
At least one CV risk	1.65 (1.00–2.72)	0.052
Aspirin	1.07 (0.67–1.69)	0.787
Phlebotomy	0.88 (0.57–1.37)	0.567
Hydroxyurea	0.85 (0.56–1.31)	0.465

*HR* hazard ratio, *CI* confidence interval, *CV* cardiovascular.

### Increased discriminatory ability of NLR when added to standard risk factors of venous thrombosis

To quantify the discriminatory prognostic capacity of NLR, we calculated the Harrell’s C-statistic of two nested models, one that included conventional standard risk classification only (i.e., age and previous thrombosis) and one that added to the latter the NLR value above 5, looking for any incremental value of the statistic. The probability that predictions and outcomes are concordant measured by the C-statistic for the first model was 59.24%, while after adding the NLR variable, it increased to 65.09%.

**Fig. 2:**
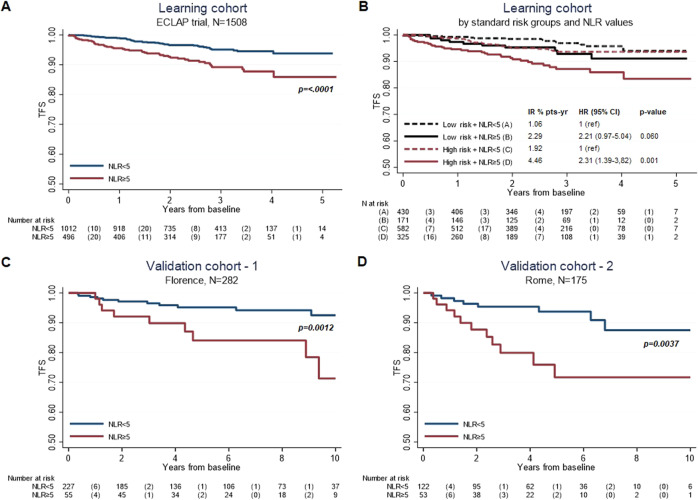
Venous thrombosis-free survival by NLR values. Kaplan–Meier venous thrombosis-free survival estimates according to categories of NLR values (<5 or ≥5) in the learning cohort of ECLAP trial (overall [**A**] and stratified by standard risk categories [**B**]), and in two external validation cohorts of contemporary PV patients (Florence [**C**] and Rome [**D**]).

Kaplan–Meier curves of time-to-venous thrombosis probability stratified by either conventional risk classification or values of NLR are plotted in Fig. 2B. Of note, the relative risk, in both low- and high-standard risk group, was almost doubled in the presence of NLR ≥ 5.

### Validation

The correlation between NLR values and venous thrombosis found in our learning cohort (Fig. 2A) was validated in two Italian independent external cohorts (Florence, *n* = 282 and Rome, *n* = 175) of contemporary PV patients, whose clinical characteristics are presented in Table 5. Kaplan–Meier venous thrombosis-free survival estimates confirmed the significant prognostic effect of NLR ≥ 5 in both external cohorts (Fig. 2C, D).

**Table 5:** Baseline characteristics of the Florence and Rome external validation cohorts.

	Florence cohort *N* = 282	Rome cohort *N* = 175
Males/Females, *n* (%)	164/118 (58/42)	96/79 (54.9/45.1)
Age, years, median (95% CI)	64 (18–91)	61 (27–92)
*Blood counts, median (range)*		
Hemoglobin g/dL	17.6 (15.6–23.5)	16.6 (12.2-22.2)
Platelets x10^9^/L	476 (190–890)	468 (99-1320)
WBC x10^9^/L	9.4 (4.5–18.9)	9.7 (3.7–44.4)
Neutrophils %		71.9 (43.4–91.3)
Lymphocytes %		18.7 (5.3–44.5)
NLR	3.5 (0.6–16.3)	3.83 (0.98–16.95)
At least one CV risk factor, *n* (%)	164 (58)	103 (58.9)
Previous arterial thrombosis, *n* (%)	37 (13.1)	17 (9.7)
Previous venous thrombosis, *n* (%)	33 (11.7)	16 (9.1)
*Treatments, n (%)*		
Phlebotomy	282 (100)	151 (86.3)
Hydroxyurea	175 (62)	116 (66.3)

*WBC* white blood cells, *NLR* neutrophil/lymphocyte ratio, *CV* cardiovascular.

## Discussion

In this series of patients with PV, we sought to investigate for the first time whether the baseline ratio of circulating neutrophils to lymphocytes (NLR) could be a marker of thrombosis as reported in many studies in the general population [23]. To this purpose, we examined 1,508 patients enrolled in the ECLAP database in which a large number of thrombotic events (venous and arterial) were registered and objectively diagnosed as required in this randomized placebo-controlled clinical trial [7].

In univariate analysis, we found that the absolute number of neutrophils and lymphocytes was on average respectively higher (median: 6.9 × 10^9^/L, *p* = 0.022) and lower (median: 1.3 × 10^9^/L, *p* = 0.002) in patients with incident venous thrombosis, overall resulting in a higher NLR ratio. The most evident finding was the linear association between the absolute number of lymphocytes and the risk of events expressed by the log of HRs in the GAM models. We found that the category of patients presenting at baseline with lymphocyte values less than 2 × 10^9^/L had an increased risk of venous thrombosis with HR greater than one, whereas the HRs for venous thrombosis progressively decreased as the lymphocyte counts increased. These changes are consistent with an opposite prognostic tendency of neutrophils, whose increments showed an increasingly higher trend of risk (HR = 1.77, *p* = 0.160).

As a result, the NLR integrates these two opposing trends, being more precise than individual counts alone to predict venous thrombosis, as shown by the narrower confidence intervals. As recently reported [16], we also failed to show that baseline total leukocyte and platelet counts correlate with either venous or arterial thrombosis and similar results were described in a previous analysis of the same ECLAP study [15].

In a multivariate Cox proportional-hazard model using NLR cutoff equal to five, found to most efficiently discriminate against the highest risk categories, we found that this cutoff value was associated with major venous events (HR = 2.18), independently of age and previous venous thrombosis, and this was confirmed in the sensitivity analysis after excluding minor venous thrombosis. On the contrary, NLR did not show any significant association with arterial events that were predicted by previous arterial event (*p* = 0.01) and age ≥65 years.

Interestingly, the prognostic discriminatory power of conventional risk factors for venous thrombosis was increased in the presence of an elevated NLR as demonstrated by the values of the C-statistic increasing from 59% to 65%, in either low- or high-risk PV groups. This finding could therefore suggest the value of incorporating this inflammatory biomarker in a scoring system for venous thrombosis in PV.

It will be interesting to investigate the relationship between NLR values and the allelic burden of *JAK2*V617F, which has recently been reported to be correlated with venous thrombosis [24]. Furthermore, it is worth noting that the association of absolute values of circulating neutrophil, lymphocyte, and monocyte with venous but not with arterial thrombosis was also found in essential thrombocythemia, further corroborating the role of inflammation in thrombogenesis of MPN.

We are fully cognizant of the limitations of the current study considering that this is a post hoc analysis of a clinical trial using data that had already been collected for other purposes and referred to patients diagnosed with PVSG diagnostic criteria [25]. However, we underscore that the study population was not selected, as all comers with PV were included, and the results were validated by two external cohorts that had substantially the same clinical characteristics as the original learning series. It is worth noting that the two validation cohorts included only patients with WHO-2016 diagnosis and the NLR test was calculated at diagnosis.

In conclusion, these clinical findings support recent biological concepts that innate immune cells contribute to venous thromboembolism [26]. Indeed, neutrophils forming extracellular traps promote thrombus accretion by presenting proteases, coagulation factors, and serving as a scaffold for cell attachment and fibrin polymerization. Although the role of lymphocytes in acute thrombogenesis is unclear, lymphocytes modulate innate immune-cell recruitment and activity during thrombus resolution. A recently described, specialized subset of T-reg lymphocytes accumulate in venous thrombi and are essential to clot resolution [27–29]. These mechanistic studies point toward active cross-talk between the innate and adaptive immune systems in venous thrombosis, bolstering the biologic plausibility of our clinical study positing NLR as an easily available biomarker of venous thrombosis risk in PV.

## Supplementary information

Reproducibility checklist
